# Genistein and Vanadate Differentially Modulate Cortical GABA_A_ Receptor/ATPase Activity and Behavior in Rats via a Phenol-Sensitive Mechanism

**DOI:** 10.3390/ijms26125731

**Published:** 2025-06-15

**Authors:** Sergey A. Menzikov, Danila M. Zaichenko, Aleksey A. Moskovtsev, Sergey G. Morozov, Aslan A. Kubatiev

**Affiliations:** Institute of General Pathology and Pathophysiology, 8, Baltiyskaya St., Moscow 125315, Russia; danilamihailovich@mail.ru (D.M.Z.); bioinf@mail.ru (A.A.M.); biopharm@list.ru (S.G.M.); aslan.kubatiev@gmail.com (A.A.K.)

**Keywords:** genistein, vanadate, synaptoneurosomes, transport, GABA_A_Rs/ATPase, recombinant α2β3γ2 GABA_A_Rs, phenol

## Abstract

Although some GABA_A_ receptor subtypes are involved in both the passive permeability of anions and the ATP-dependent recovery of neuronal anion concentrations, the molecular mechanisms that ensure the coordination of passive and active transport processes remain unclear. Here we used fluorescence measurements to investigate the role of genistein (tyrosine kinase inhibitor) and vanadate (tyrosine phosphatase and ATPase inhibitor) in modulating GABA_A_R-mediated [Cl^−^]_i_/[HCO_3_^−^]_i_ changes and ATPase activity in rat cortical neurons and HEK 293FT cells expressing the heteropentameric α2β3γ2 GABA_A_R isoform. We found that genistein plays an important role in the inhibition of passive GABA_A_R-mediated Cl^−^ influx and Cl^−^ATPase activity, whereas vanadate plays an important role in the inhibition of Cl^−^, HCO_3_^−^ATPase activity and ATP-dependent recovery of [HCO_3_^−^]_i_ via changes in the formation of the phosphorylated intermediate. The effect of blockers was significantly restored in the presence of phenol. In behavioral experiments, the administration of phenol has been established to induce tremors and head twitching in rats, with the involvement of GABA_A_R/ATPase in these behavioral responses. Genistein can reduce the adverse effects of phenol, thereby confirming the interaction of these chemicals when binding to binding receptor sites. While our data demonstrate the opposing roles of genistein and vanadate in modulating GABA_A_R/ATPase function in a bicarbonate-dependent manner. Such multidirectional systems are considered to be bistable elements involved in the regulatory mechanisms of synaptic plasticity.

## 1. Introduction

Phosphorylation has been identified as playing a pivotal role in all cellular signaling pathways, including those associated with post-translational modifications of plasma membrane structures (including ion channels or transporters). These modifications result in alterations to the structural conformation and function of the proteins concerned [1]. Maintenance of phosphorylation is characterized by the opposite transfer (input/output) of γ-phosphate (P_i_) with adenosine triphosphate (ATP) to target amino acid residues, which are catalyzed by protein kinases (PKs) and protein phosphatases (PPs), respectively. Phosphorylation and dephosphorylation are highly dynamic processes targeted at specific molecular substrates. Questions regarding how these sites are recognized and the mechanisms that coordinate the balance of the enzymes involved are significant for elucidating their physiological functions and for the treatment of diseases [2].

**Figure 1:**
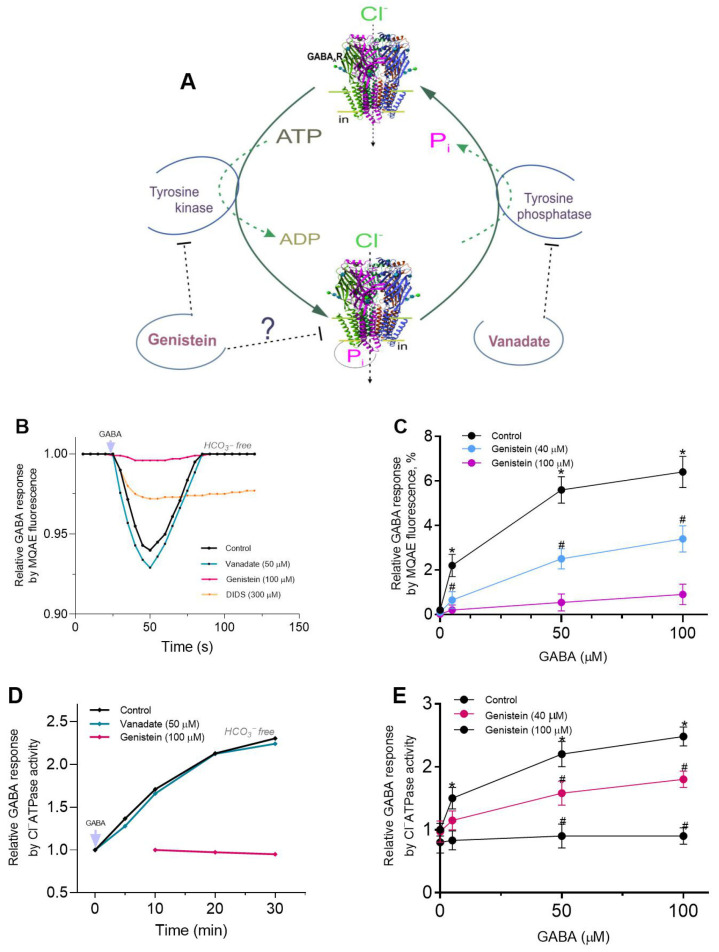
Genistein, in contrast to vanadate, inhibits GABA-mediated Cl^−^ influx and Cl^−^ATPase activity. (**A**) Sketch of prevailing antagonistic regulation of GABA_A_Rs by genistein and vanadate. Genistein decreases the receptor function via inhibition of the PTKs’ activity or directly inhibits the GABA_A_Rs, whereas vanadate elevates the receptor function via inhibition of PTPs. (**B**) Corresponding temporary MQAE fluorescence changes for SNs recording in response to 50 μM GABA application, in the absence or presence of 50 μM vanadate, 100 μM genistein or 300 μM DIDS in HCO_3_^−^-free experimental medium. (**C**) Bar chart showing relative GABA (0, 5, 50 or 100 μM) responses for SNs by MQAE fluorescence changes before and after 100 μM genistein application. (**D**) Corresponding temporary changes for Cl^−^ATPase activity recording in response to 50 μM GABA application, in the absence or presence of 50 μM vanadate or 100 μM genistein in an experimental medium. (**E**) Bar chart showing relative GABA (0, 5, 50 or 100 μM) responses by Cl^−^ATPase activity changes before or after 100 μM genistein application. Data are presented as mean value ± SEM, *p* < 0.05. * significant to baseline; ^#^ significant to control. Statistical differences were determined by a two-tailed Student’s unpaired *t*-test for data with equal variances, which were assessed as normally distributed with the Shapiro–Wilk test.

γ-Aminobutyric acid type A receptors (GABA_A_Rs) are members of the pentameric ligand-gated ion channels (pLGICs) family that mediate rapid neurotransmission in the nervous system [3]. Their dysfunction is associated with neurodegenerative and psychiatric processes, including memory, epilepsy, tremor, anxiety, and sedation [4]. Several studies have demonstrated the critical role of protein tyrosine kinases (PTKs) [5] and protein tyrosine phosphatases (PTPs) [6] in the regulation of pLGICs (including GABA_A_Rs) (see Figure 1A). As demonstrated by several reports, genistein (a PTK inhibitor) has been shown to depress GABA-mediated Cl^−^ inflow in neuronal and recombinant GABA_A_Rs [6,7]. Conversely, vanadate (a PTP and P-type ATPase inhibitor) has been observed to produce a modest increase in GABA_A_R activity [8,9,10]. Genistein, in conjunction with other isoflavones, has been shown in some works to exert a direct inhibitory effect on GABA_A_Rs, independent of kinase involvement [11,12]. Notwithstanding the fact that the molecular mechanisms of direct genistein action on receptor activity remain unclear, it has been proposed that this chemical acts as an estrogen agonist via allosteric interaction with the receptor [4]. While there are multiple pathways by which phenols can interact with membrane structures, the predominant pathway is the formation of hydrogen bonds. Specifically, the OH groups in phenol chemicals have the capacity to interact with receptors via the formation of H-bonds with amino acid residues (for example, the phenolic hydroxyl group of tyrosine residue) [4,13]. Conversely, certain authors have demonstrated that vanadate engages in an interaction with tyrosine or phenol, resulting in the esterification of hydroxyl groups, thereby yielding vanadate esters [14]. It was hypothesized that, given the established importance of phenol in the modulation of GABA_A_R activity [15,16], genistein and vanadate could regulate receptor function via a phenol-sensitive mechanism.

GABA_A_Rs in mature neurons are the primary inhibitory receptors, a consequence of the permeability of chloride (Cl^−^) ions through the ion channel pore [17]. However, under certain circumstances (for example, intensive or prolonged activation), GABAergic signaling has been demonstrated to exhibit depolarization/excitatory responses. This is due to the formation of a high intracellular chloride concentration ([Cl^−^]_i_) and outflow of not only Cl^−^ but also bicarbonate (HCO_3_^−^) ions via receptor pore [18,19]. The pentameric assemblies of GABA_A_Rs are composed of nineteen possible subunits, of which the most common subtypes observed in the vertebrate brain have a 2α:2β:1γ composition [20]. As demonstrated in some studies, the sites for phosphorylation by PTKs and dephosphorylation by PTPs have been identified in β subunits [8,9]. Moreover, an accumulation of data has demonstrated the unique role of the β3 subunit in the manifestation of receptor properties at both the molecular and circuit level (for example, ionic/ligand selectivity, pharmacological characteristics, etc.) [20,21]. Recent studies have demonstrated that the GABA_A_R β3 subunit possesses a phenol-sensitive ATPase, which provides the transition of GABA_A_Rs from a desensitization to resensitization state [22,23]. As genistein and vanadate target sites implicated in ATP-dependent processes within the receptor structure, it was imperative to investigate their impact on passive anion permeability via ion channel pore, as well as on GABA_A_R-coupled ATPase activity. It is evident that the physiological level of genistein is minimal (i.e., ≤5 µM), and it is able to act only as a modulator at the endoplasmic reticulum [11,12]. Nevertheless, in instances where a diet may comprise high doses of isoflavones (≤20 µM) in the blood, genistein has been demonstrated to mediate neuroprotection in cases of status tremor, seizures and Alzheimer’s disease [24,25]. It is improbable that the direct inhibition of GABA_A_Rs by genistein (with effective concentrations of greater than 70 µM) will occur in humans at dietary concentrations of genistein that are relevant to humans [26,27,28]. However, the molecular mechanisms through which genistein exerts its effects on neurodegenerative processes remain to be fully elucidated [16,26]. Given the role of GABA_A_R/ATPase effect in phenol-induced behavioral symptoms, it seemed important to investigate genistein action on phenol-induced tremor and seizures.

In this work, we used the fluorescence measurements (by dyes for Cl^−^ and HCO_3_^−^) to evaluate the role of genistein and vanadate in the modulation of the GABA_A_R-mediated [Cl^−^]_i_/[HCO_3_^−^]_i_ changes and ATPase activity in the cortex synaptoneurosomes (SNs) of rats and HEK 293 FT cells expressing the heteropentameric α2β3γ2 GABA_A_R subtype isoform. Here, we illustrated that genistein plays an essential role in the inhibition of the GABA_A_R-mediated Cl^−^ influx and Cl^−^ATPase activity in the neurons in an HCO_3_^−^-free environment, whereas vanadate plays an important role in the inhibition of these processes in the presence of a physiologic bicarbonate concentration. We showed that genistein and vanadate produce an increase in phosphoprotein formation at the membrane-bound receptor but lead to the recovery of anion-induced dephosphorylation. In addition, we showed the role of genistein in producing a decrease in phenol-induced neurological disorders in rats. Overall, our data reveal the essential role of bicarbonate in coordinating the effects of genistein and vanadate on cortical GABA_A_R/ATPase activity via a phenol-sensitive mechanism.

## 2. Results

### 2.1. Genistein, in Contrast to Vanadate, Inhibits GABA-Mediated Cl^−^ Influx and Cl^−^ATPase Activity

Upon agonist binding, the transmembrane pore of the pLGICs (including GABA_A_Rs) quickly opens to enable the selective flow of permeant ions across the plasma membrane, thereby affecting cell excitability [17]. During sustained binding of the agonist, most will gradually go into a closed or desensitized state refractory to activation [3,29]. A fluorescence-based method, in contrast to a patch-clamp method, does not directly measure ionic current and only measures the ion-concentration-dependent changes in fluorescence signals due to ionic flux; it can detect the conformational changes in receptors [30]. Moreover, ligand-dependent fluorescence changes can determine channel activation, inhibition and modulation but cannot control the membrane potential as in electrophysiological assays. Here, cortical SNs, which previously were loaded with a Cl^−^-sensitive fluorescent dye MQAE (N-(Ethoxycarbonyl-methyl)-6-Methoxyquinolinium Bromide), manifest the bicuculline-sensitive GABA_A_R-mediated Cl^−^ influx in response to the application of GABA (50 μM) in HCO_3_^−^-free experience medium with a maximum peak in MQAE fluorescence changes of 5.6 ± 0.6%, (*n* = 5) (Figure 1B).

Although the biological effects of genistein in *in vivo* and *in vitro* cellular activities have been reported to occur over a wide µM range of concentrations [28], several studies reported that genistein (50–100 μM) inhibits the GABA-activated currents in native neurons [11,12] or muscimol-stimulated ^36^Cl uptake in cortical microsacs [7]. In our study, genistein (100 µM) almost completely inhibits GABA-mediated Cl^−^ influx into SNs, whereas in the presence of vanadate (50 μM), the GABA_A_R-mediated Cl^−^ influx is increased slightly (7.1 ± 0.2%, *n* = 5). These data are comparable to literature data [11,12]. While some studies demonstrated that the application of the vanadate (100 µM) caused a low increase in the GABA-evoked Cl^−^ current in the neurons [8,9]. Preliminary electrophysiological studies have shown that with increasing agonist concentration (1–1000 μM), there is an increase in I_GABA_ by recombinant α2β3γ2L GABA_A_R, with maximum effect in the presence of 1000 μM [31]. In the studied range (5, 50 or 100 μM) of GABA concentrations, there is also an increase in Cl^−^ influx in the neurons (Figure 1C). As shown in Figure 1C, genistein inhibition of GABA_A_R-mediated Cl^−^ influx is non-competitive with little dependence on the agonist concentration. The obtained data demonstrate bell-shaped type kinetics during the time course of GABA responses by MQAE. Given that the bicarbonate concentration in the buffer is 49 mg/L (0.59 mM), we hypothesized that this kinetic behavior might be related to the restoration of anion homeostasis. To clarify whether a system (similar to the Cl^−^/HCO_3_^−^ exchanger) is involved in the restoration of anion homeostasis following receptor activity [32] or resensitization state [33], we added 4,4′-diisothiocyanatostilbene-2,2′-disulfonic acid (DIDS) to the experience medium [34]. DIDS (300 μM) significantly reduced the effect of [Cl^−^]_i_ recovery after mediator application (Figure 1B).

Previously, we showed that activity of GABA_A_R-coupled ATPase in the cortical membranes from rat brain consists of Cl^−^ATPase and Cl^−^, HCO_3_^−^ATPase form activities [22,23]. Also, it was established that GABAergic ligands (agonists and antagonists) can modulate the ATPase activity of the native and recombinant GABA_A_R subtypes [23]. In this study, the bicuculline-sensitive Cl^−^ATPase activity in the plasma membranes (PMs) from the cerebral cortex was 330.0 ± 19.0 nmol P_i_⋅min^−1^⋅mg^−1^, and GABA (50 μM) activated Cl^−^ATPase activity during 0−30 min preincubation with maximum effect at 30 min. As shown in Figure 1D, genistein (100 μM) inhibits the activating effect of the mediator on enzyme activity, whereas vanadate did not produce any effects on such enzyme activity. Inhibition of Cl^−^ATPase activity by genistein is non-competitive with little dependence on the mediator concentration (0, 5, 50 or 100 μM) (Figure 1E).

### 2.2. Bicarbonate Changes the Effect of Genistein and Vanadate on the GABA-Mediated Cl^−^ Influx and Cl^−^, HCO_3_^−^ATPase Activity

**Figure 2:**
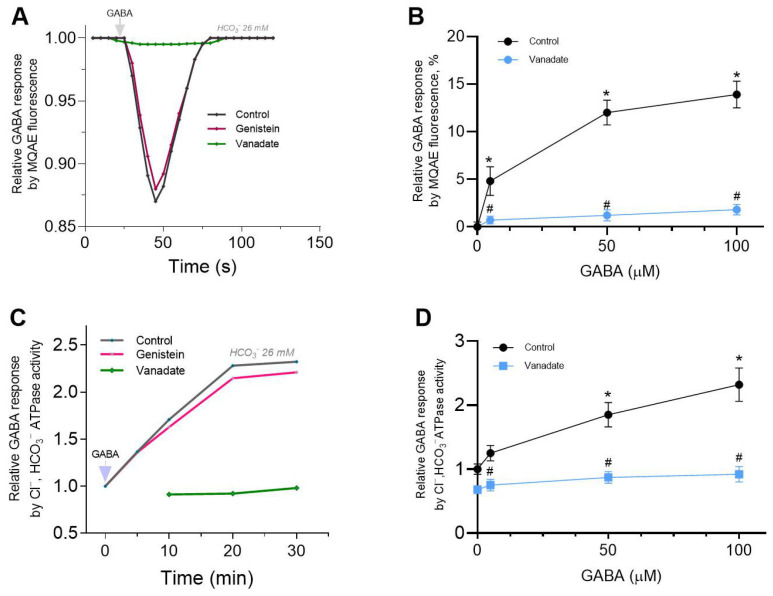
Bicarbonate prevents the genistein effect on GABA-mediated Cl^−^ influx and Cl^−^, HCO_3_^−^ATPase activity. (**A**) Corresponding temporary MQAE fluorescence changes for SNs recording in response to 50 μM GABA application, in the absence and presence of 50 μM vanadate or 100 μM genistein, and in the presence of 26 mM HCO_3_^−^ in an experimental medium. (**B**) Bar chart showing relative GABA (0, 5, 50 or 100 μM) responses for SNs by MQAE fluorescence changes before and after 50 μM vanadate application. (**C**) Corresponding temporary changes for Cl^−^, HCO_3_^−^ATPase activity of the PMs recording in response to 50 μM GABA application, in the absence or presence of 50 μM vanadate or 100 μM genistein in an experimental medium. (**D**) Bar chart showing relative GABA (0, 5, 50 or 100 μM) responses by Cl^−^,HCO_3_^−^ATPase activity changes before and after 50 μM vanadate application. Data are presented as mean value ± SEM, *p* < 0.05. * significant to baseline, ^#^ significant to control. Statistical differences were determined by a two-tailed Student’s unpaired *t*-test for data with equal variances, which were assessed as normally distributed with the Shapiro–Wilk test.

Previous research demonstrated that physiological concentrations of HCO_3_^−^ (~26 mM) produced an increase in the GABA_A_R-mediated Cl^−^ influx in the neurons [23]. In our study, SNs in the presence of 26 mM HCO_3_^−^ showed an increase in Cl^−^ influx in response to 50 μM GABA application with a maximum peak of fluorescence changes of 13.9 ± 0.4% (*n* = 5), as shown in Figure 2A. Genistein (100 μM) did not produce significant effects on GABA-mediated fluorescence changes (Figure 2A), while in the presence of vanadate (50 μM), the GABA_A_R-mediated Cl^−^ influx into neurons did not appear.

Moreover, the GABA concentration-dependent responses (0, 5, 50 or 100 μM) revealed that vanadate completely eliminated the GABA_A_R-mediated fluorescence changes and was non-competitive with minimal dependence on the mediator concentration, as shown in Figure 2B.

For the purpose of comparison with Cl^−^ATPase, we studied the Cl^−^, HCO_3_^−^ATPase form and the activity, which is 692.0 ± 24.0 nmol P_i_⋅min^−1^⋅mg^−1^. GABA (1–50 μM) activated Cl^−^, HCO_3_^−^ATPase activity with a maximum effect at 50–100 μM (Figure 2C). Application of genistein (100 µM) did not produce changes in the GABA’s effect on enzyme activity, whereas vanadate (50 μM) completely inhibited it. As shown in Figure 2D, inhibition of Cl^−^, HCO_3_^−^ATPase activity by vanadate had little dependence on the mediator concentration.

### 2.3. Vanadate and Genistein Differentially Modulate the GABA_A_R-Mediated pH_i_ Recovery and ATPase Activity

**Figure 3:**
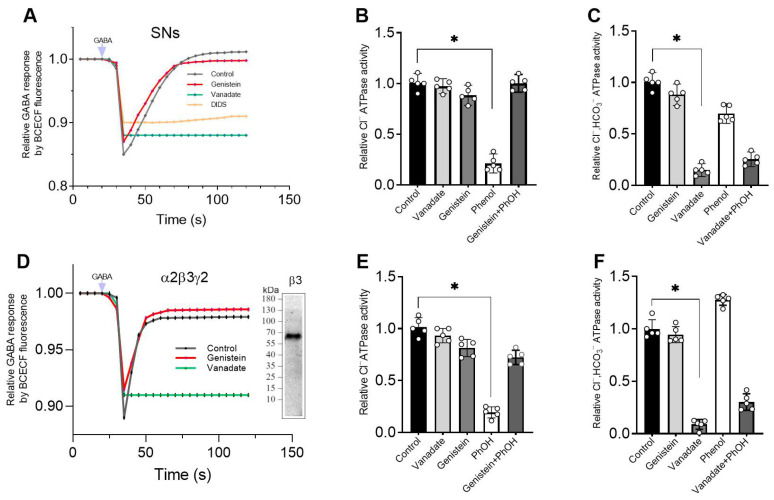
Vanadate and genistein differently modulate GABA_A_R-mediated pH_i_ recovery and ATPase activity. (**A**) Corresponding temporary BCECF fluorescence changes for SNs recording in response to 50 μM GABA application, in the absence and presence of 50 μM vanadate, 100 μM genistein or 300 μM DIDS, and in the presence of 26 mM HCO_3_^−^ in an experimental medium. (**B**) Bar chart showing the relative Cl^−^ATPase activity changes before and after 50 μM vanadate, 100 μM genistein, 2 mM PhOH or 100 μM genistein +2 mM PhOH application: Student’s unpaired *t*-test, *p* < 0.0001, t = 16.78 (*n* = 5). (**C**) Bar chart showing the relative Cl^−^, HCO_3_^−^ATPase activity changes before and after 100 μM genistein, 50 μM vanadate, 2 mM PhOH or 50 μM vanadate +2 mM PhOH application: Student’s unpaired *t*-test, *p* < 0.0001, t = 21.29 (*n* = 5). (**D**) Corresponding temporary BCECF fluorescence changes in HEK 293FT cells expressing the heteropentameric α2β3γ2 GABA_A_R subtype and recording in response to 50 μM GABA application, in the absence or presence of 50 μM vanadate or 100 μM genistein in an experimental medium. Western blot analysis of the binding of VLPs with antibody against the GABA_A_R β3 subunit after expression of DNA GABA_A_R subtype in HEK 293FT cells. (**E**) Bar chart showing the relative Cl^−^ATPase activity changes in the plasma membranes from HEK 293FT cells expressing the heteropentameric α2β3γ2 GABA_A_R subtypes before and after 50 μM vanadate, 100 μM genistein, 2 mM PhOH or 100 μM genistein +2 mM PhOH application: Student’s unpaired *t*-test, *p* < 0.0001, t = 17.94 (*n* = 5). (**F**) Bar chart showing the relative Cl^−^, HCO_3_^−^ATPase activity changes in HEK 293FT cells expressing the heteropentameric GABA_A_R subtypes before and after 100 μM genistein, 50 μM vanadate, 2 mM PhOH or 50 μM vanadate +2 mM PhOH application: Student’s unpaired *t*-test, *p* < 0.0001, t = 20.3 (*n* = 5). Data are presented as mean value ± SEM, *p* < 0.05. * significant to control. Statistical differences were determined by a two-tailed Student’s unpaired *t*-test for data with equal variances, which were assessed as normally distributed with the Shapiro–Wilk test.

Previous studies demonstrated that the receptor channel is permeable not only for Cl^−^ ions but also for HCO_3_^−^ after prolonged GABA exposure that leads to their outward flow with a consequent gradual recovery [22]. As illustrated in Figure 3A, the SNs, which previously were loaded with a pH-sensitive fluorescent dye BCECF (2′,7′-Bis-(2-Carboxyethyl)-5-(and-6)-Carboxyfluorescein, Acetoxy methyl Ester), showed a rapid decrease in intracellular pH (pH_i_) in response to GABA (50 μM) addition with a maximum peak in fluorescence change of 15.0 ± 0.5% (*n* = 5) and a subsequent recovery. Genistein (100 μM) did not produce any effects on GABA-mediated fluorescence changes, whereas in the presence of 50 μM vanadate, the GABA-mediated pH_i_ recovery did not appear (Figure 3A). To confirm the role of Cl^−^/HCO_3_^−^ exchange transport in the manifestation of bell-shaped kinetics, 300 μM DIDS was added to the incubation medium. As shown in Figure 3A, DIDS eliminates this bell-shaped effect.

To assess the role of genistein and vanadate in GABA_A_R/ATPase modulation of SNs, we studied their effect on Cl^−^ATPase and Cl^−^, HCO_3_^−^ATPase activities in the plasma membranes. The Cl^−^ATPase activity is 337.8 ± 26.0 nmol^−1^ P_i_ min^−1^ mg^−1^ (Figure 3B). The addition of genistein (100 μM), in contrast to vanadate (50 μM), completely inhibited the Cl^−^ATPase activity. Phenol (2 mM) restores the inhibition action of genistein on the enzyme activity (Figure 3B). While the Cl^−^,HCO_3_^−^ATPase activity is 692.0 ± 24.0 nmol^−1^ P_i_ min^−1^ mg^−1^. As shown in Figure 3C, the addition of vanadate (50 μM), in contrast to genistein (100 μM), eliminates enzyme activity. Phenol (2 mM) produced a minor recovery from the inhibiting effect of vanadate on the enzyme activity.

Previously, we demonstrated that the HEK 293 FT cells expressing the α2β3γ2 GABA_A_R subtypes are involved in GABA_A_-mediated Cl^−^ influx [10] and the GABA_A_R β3 subunit is involved in HCO_3_^−^ outflow [23]. Here, we studied the properties of the GABA_A_R-mediated HCO_3_^−^ outflux in HEK 293FT cells that expressed the α2β3γ2 GABA_A_R isoform. HEK 293 FT cells expressing the GABA_A_R β3 isoform showed one band in the VLPs, with a molecular weight of approximately 54 kDa, that bound with the antibodies against the GABA_A_R β3 subunit (Figure 3D). In the presence of 26 mM HCO_3_^−^ in the experimental medium containing HEK 293FT cells, rapid pH_i_ decreases after applications of the GABA (50 μM) with a maximum peak of fluorescence changes of 11.2% ± 0.8%, and the subsequent recovery of [HCO_3_^−^]_i_ could be observed (Figure 3D). We tested the effect of genistein (100 μM) and vanadate (50 μM) on the GABA-mediated pH_i_ changes. As shown in Figure 3D, vanadate completely inhibited the GABA_A_R-mediated HCO_3_^−^ recovery, whereas genistein did not produce any effects on fluorescence changes.

We appreciated the role of genistein and vanadate in ATPase regulation of PMs. The Cl^−^ATPase activity in the PMs from HEK 293 FT cells is 229.5 ± 15.0 nmol^−1^ P_i_ min^−1^ mg^−1^ (Figure 3E). The addition of genistein (100 μM), in contrast to vanadate (50 μM), completely inhibited the enzyme activity. Phenol (2 mM) produced a recovery of the inhibiting effect of genistein on the enzyme activity (Figure 3E). While, as shown in Figure 3F, the Cl^−^, HCO_3_^−^ATPase activity is 417.4 ± 36.0 nmol^−1^ P_i_ min^−1^ mg^−1^. The addition of vanadate (50 μM), in contrast to genistein (100 μM), decreases the enzyme activity. Phenol (2 mM) produced a minor recovery of the inhibiting effect of vanadate (50 μM) on the Cl^−^, HCO_3_^−^ATPase activity (Figure 3F).

### 2.4. Genistein in Contrast to Vanadate Modulates Rapid Phosphoprotein Formation via Phenol-Sensitive Mechanism

**Figure 4:**
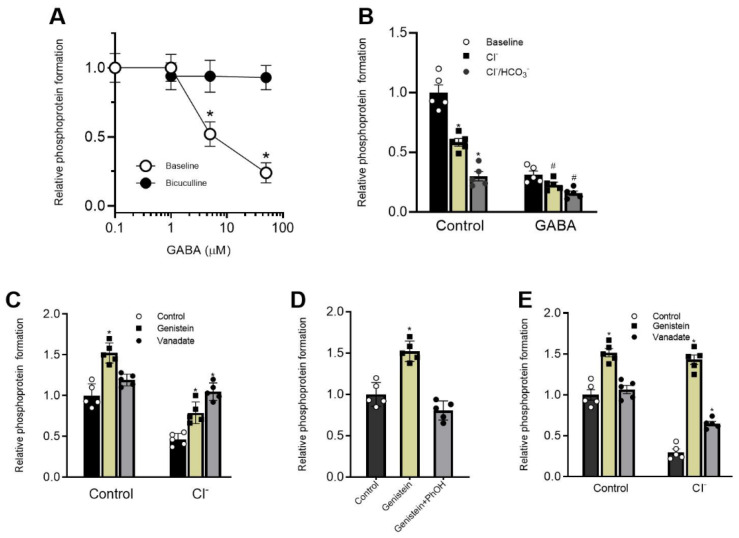
Genistein and vanadate eliminate the Cl^−^ or Cl^−^/HCO_3_^−^-induced dephosphorylation via different mechanisms. (**A**) Bar chart showing the relative phosphoprotein formation before and after 50 μM GABA application, in the absence or presence of 40 μM bicuculline in an experimental medium. (**B**) Bar chart showing the relative phosphoprotein formation before and after 25 mM Cl^−^ or 5 mM Cl^−^/25 mM HCO_3_^−^ application, in the absence or presence of 50 μM GABA: Student’s unpaired *t*-test, *p* < 0.0001, t = 20.92/t = 1,458 and t = 9.6/t = 11.34 (*n* = 5). (**C**) Bar chart showing the relative phosphoprotein formation before and after 100 μM genistein or 50 μM vanadate in the absence or presence of 25 mM Cl^−^: Student’s unpaired *t*-test, *p* < 0.0003, t = 6.137 and *p* < 0.04, t = 6.137/t = 2.4 (*n* = 5). (**D**) Bar chart showing the relative phosphoprotein formation before and after 100 μM genistein or 100 μM genistein +2 mM phenol Student’s unpaired *t*-test, *p* < 0.0003, t = 6.137 (*n* = 5). (**E**) Bar chart showing the relative phosphoprotein formation before and after 100 μM genistein or 50 μM vanadate, in the absence or presence of 5 mM Cl^−^/25 mM HCO_3_^−^: Student’s unpaired *t*-test, *p* < 0.0001, t = 6.137 and t = 19.5/t = 7.83 (*n* = 5). Data are presented as mean value ± SEM, *p* < 0.05. * significant to control or # baseline, *n* = 4. Statistical differences were determined by a two-tailed Student’s unpaired *t*-test for data with equal variances, which were assessed as normally distributed with the Shapiro–Wilk test.

The direct phosphorylation by ATP-γ-^32^P of P-type ATPases, including the GABA_A_R-coupled ATPase of interest here, requires Mg^2+^ [15]. In the present work, the membrane-bond receptor was tested for phosphorylation in the presence of [γ-^32^P]ATP and Mg^2+^ (3 mM). A maximum incorporation of ^32^P (3750.0 ± 102 pmol ^32^P mg protein) into membrane preparation occurred after 2 min incubation with 90 μmol ATP-γ-^32^P in the presence of 3 mM Mg^2+^. After application of NH_2_OH (10 mM) or buffer (pH 10.0), the phosphoprotein formation does not appear. In order to establish belonging to the GABA_A_R structure, we added GABA (50 μM) to the experience medium. GABA induces a decrease in the phosphoprotein formation by more than two times (Figure 4A). The effect of the agonist does not appear in the presence of bicuculline (40 μM), indicating the receptor-dependent way of action.

In the literature, it was shown that for ATPases, the P-type family is characteristically the dephosphorylation by transporting ions [15]. In our study, Cl^−^ or Cl^−^/HCO_3_^−^ with various efficacy reduced phosphoprotein formation, and their effect was eliminated in the presence of 50 μM GABA (Figure 4B). Here, we also tested the effect of Cl^−^ and Cl^−^/HCO_3_^−^ on the phosphoprotein formation in the absence and presence of genistein and vanadate. In the absence of anions, genistein (100 μM), in contrast to vanadate (50 μM), increases the baseline phosphoprotein formation by 1.5 times (Figure 4C). In addition, the application of genistein eliminates the Cl^−^-induced baseline dephosphorylation. Previously, we showed that phenol increased the phosphoprotein formation in the low concentrations but inhibited it in high concentrations [15]. Here, phenol (2 mM) effectively restores the genistein-induced baseline phosphoprotein increase (Figure 4D). While, as shown in Figure 4E, the Cl^−^, HCO_3_^−^-induced dephosphorylation was inhibited by vanadate but was not sensitive to genistein.

### 2.5. Genistein Diminishes Phenol-Induced Behavioral Symptoms in Rats

Genistein is not a natural endogenous product and can potentially have a negative effect on the GABA_A_Rs’ function only in high concentrations and, as a result, produce the disturbance of the balance of synaptic inhibition/excitation [27,35]. While research in recent decades has shown the positive effect of genistein and other polyphenols for a number of neurological disorders [4]. Specifically, the application of genistein significantly eliminated seizure activity in rodents [36]. Moreover, Folarin and co-authors demonstrated that polyphenols have a positive motor coordinative effect on the phenol-mediated essential tremors in a mouse model [37]. In other studies, genistein and other flavonoids (for example, resveratrol and others) have also shown significant anti-seizure activity [24,25,28].

**Figure 5:**
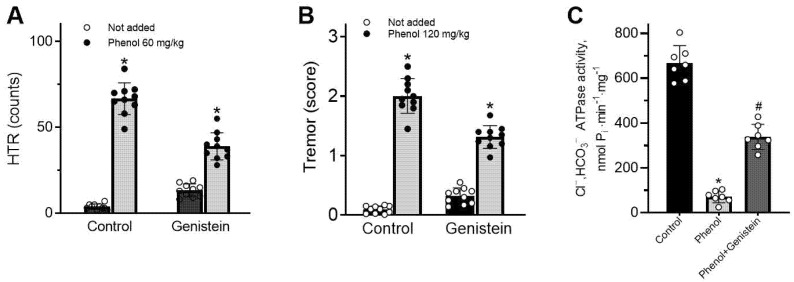
Neuroprotective effect of genistein at manifestation of phenol-induced behavioral disorders. (**A**) The magnitude of HTR in rats after injection of phenol (60 mg/kg) in the absence and in the presence of genistein (10 mg/kg): Student’s unpaired *t*-test, *p* < 0.0001, t = 21.25 and t = 7.213 (*n* = 10). (**B**) The magnitude of tremor in rats after injection of phenol (120 mg/kg) in the absence and in the presence of genistein (10 mg/kg): Student’s unpaired *t*-test, *p* < 0.0001, t = 20.29 and t = 7.213 (*n* = 10). (**C**) The Cl^−^/HCO_3_^−^-ATPase activity in brain rats after injection of phenol (120 mg/kg) in the absence and presence of genistein (10 mg/kg): Student’s unpaired *t*-test, *p* < 0.0001, t = 19.19 and t = 9.056 (*n* = 7). Data are presented as mean value ± SEM, *p* < 0.05. * significant to not added or # control. Statistical differences were determined by a two-tailed Student’s unpaired *t*-test for data with equal variances, which were assessed as normally distributed with the Shapiro–Wilk test.

In order to study the role of genistein in reducing neurological disorders, we investigated its effects on the manifestation of phenol-induced HTR and tremors in rats. Previous studies have demonstrated that phenol in the range of doses (20–160 mg/kg) induces changes in behavioral responses (including head twitching responses (HTR), tremors and seizure activity) in rodents [15]. Here, to manifest HTR or tremor symptoms in rats, we applied phenol in doses of 60 and 120 mg/kg, respectively. As shown in Figure 5A,B, no change was noted in HTR and tremor in control (saline-injected) rats. They are observed as HTR and tremors are observed as a result of phenol injection in doses of 60 and 120 mg/kg, respectively. Injections of genistein (10 mg/kg) together with phenol (60 and 120 mg/kg) eliminated the magnitude of phenol-induced HTR or tremor by 42% or 35%, respectively (Figure 5A,B). As shown in Figure 5C, the control rats manifest the Cl^−^, HCO_3_^−^ATPase activity by 692.0 ± 24 nmol P_i_⋅min^−1^⋅mg^−1^. After injection of phenol (120 mg/kg), the Cl^−^, HCO_3_^−^ATPase activity was diminished to 72.0 ± 3.0 nmol P_i_⋅min^−1^⋅mg^−1^. Simultaneously, brain Cl^−^, HCO_3_^−^ATPase activity has been substantially reversed (47%) after a combined injection of phenol (120 mg/kg) with genistein (10 mg/kg) (Figure 5C).

## 3. Discussion

In our study, increasing the time interval resulted in a decrease in GABA responses, similar to the desensitizing state observed in receptors during electrophysiological studies [31]. However, this is not entirely typical of such a process, since desensitization of the receptor would lead to a sustained decrease in fluorescence in the absence of anion exchanger activity. Probably, such kinetic behavior is also not related to disruption of cellular structure and the subsequent release of the dye into the extracellular environment (Supplement Data, Figure S1). At the same time, the literature data demonstrate that stilbene derivatives (e.g., DIDS or SITS) inhibit Cl^−^/HCO_3_^−^ exchange transport carried out by various membrane structures when present at concentrations of 100–300 μM [34]. Here, in the presence of a DIDS, the bell-shaped kinetics are significantly leveled out (see Figure 1A and Figure 3A), which suggests that the Cl^−^/HCO_3_^−^ transport process is involved in restoring anion homeostasis after receptor activation.

Genistein, targeting the ATP binding site or the substrate binding site, appeared to antagonize responses to GABA. Concentration-response curves were noncompetitively depressed, suggesting that competition at the agonist binding site between GABA and genistein is unlikely. Whereas vanadate slightly activates the GABA-mediated Cl^−^ influx into neurons. These data are comparable to those in the literature [11,12]. In particular, it was demonstrated that vanadate results in a modest increase in the GABA-induced Cl^−^ current in neurons [8,9]. Both types of inhibitor appeared to block GABA responses via different mechanisms. The data obtained in this work also indicate that genistein directly inhibits GABA-mediated chloride influx and chloride ATPase activities in neurons, assuming that no PTKs are involved in its action, only PPs. The way these chemicals act is affected by the presence of a physiological concentration of bicarbonate. Specifically, vanadate inhibits GABA_A_R-mediated Cl^−^ inflow into neurons in the presence of HCO_3_^−^ but does not affect this process in their absence. Thus, genistein and vanadate have contrary effects on GABA_A_R/ATPase activities in a bicarbonate-dependent manner. Moreover, these data indicate not only a coordinating role for HCO_3_^−^ in the differential manifestation of genistein and vanadate actions on receptor activity but also confirm a differential role for Cl^−^ and HCO_3_^−^ in passive or ATP-dependent permeability via ion channel pore. Data on the impact of genistein and vanadate on GABA-mediated pH_i_ changes also supports this. Specifically, vanadate completely inhibited GABA_A_-mediated HCO_3_^−^ recovery, whereas genistein had no effect on such fluorescence changes (see Figure 3D).

Here we showed that genistein’s inhibitory action on ATPase activity was significantly restored in the presence of phenol, unlike vanadate (Figure 3B,E). These data suggest that genistein’s effect on the receptor occurs via its interaction with the hydroxyl groups of amino acid residues (most likely, tyrosine) with the formation of H-bonds and, as a result, modulation of phosphoprotein formation [4,38]. Indeed, chemicals containing -OH groups in their structure can interact with receptors via H-bond formation. Specifically, some studies demonstrate that a direct interaction between phenol compounds and the aromatic hydroxyl of a tyrosine in the structure of pLGICs occurs [39]. While some authors have shown that phosphorylation regulates the activity of the proteins by creating a network of hydrogen bonds among specific amino acid residues nearby [38]. Genistein and vanadate produce an increase in the basal phosphoprotein level, suggesting that such an effect could be associated not only with inhibition of the activity of the receptor-bound phosphatases but also with modulation of the formation of the hydrogen bonds. Moreover, data received assume different mechanisms of interaction of these ligands with ATP-binding sites within the receptor molecule. However, it cannot be ruled out that phenol binds to a site different from the binding sites of the tested blockers in the β3 subunit structure, and their interaction occurs via the allosteric pathway. Indeed, the GABA_A_Rs can interact with a wide variety of phenol chemicals with diverse structures, and it is possible that the interaction with genistein, vanadate and phenol represents opportunistic binding of these agents to receptor molecules, involving the ion channel pore. The interaction of these chemicals at the receptor molecule is confirmed by the decrease in phenol-induced neuropsychiatric disorders in rats by genistein. These data are similar to literature data. For example, flavonoids have been shown to reduce phenol-induced tremor in mice [36,37].

**Figure 6:**
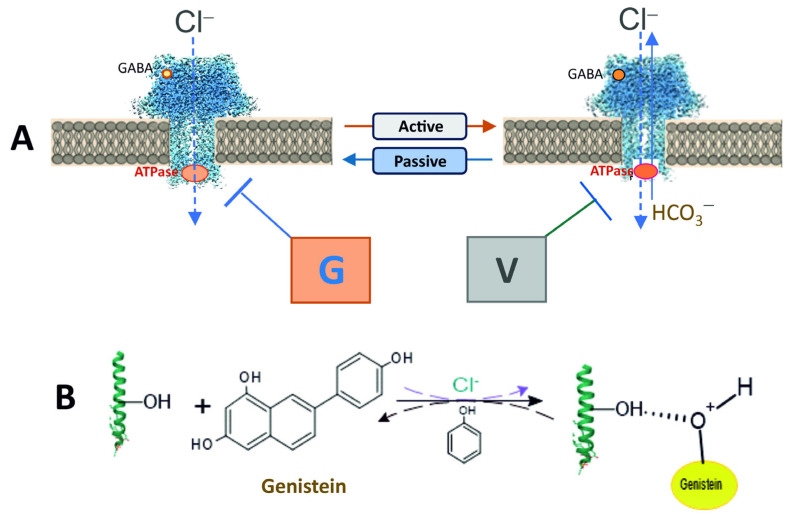
The mechanistic interpretation of direct inhibition of β3-containing GABA_A_R subtypes by genistein or vanadate. (**A**) Left sketch. Genistein inhibits both GABA_A_R-mediated [Cl^−^]_i_ changes and Cl^−^ATPase activity in the HCO_3_^−^-free experience medium. Right sketch. Vanadate inhibits both GABA_A_R-mediated [Cl^−^]_i_/[HCO_3_^−^]_i_ changes and Cl^−^, HCO_3_^−^ATPase activity in the presence of 26 mM HCO_3_^−^ in the experience medium. (**B**) The genistein action on the GABA_A_R/ATPase activity is completely restored in the presence of phenol, which suggests the interaction of genistein with the OH groups of amino acid residues (most likely, tyrosine) via the formation of H-bonds.

Overall, our data demonstrate the opposing roles of genistein and vanadate in modulating GABA_A_R/ATPase function. Such multidirectional systems are considered to be bistable elements involved in the regulatory mechanisms of synaptic plasticity [1]. In addition, our study has established an essential role for HCO_3_^−^ in regulating the action of these chemicals with different structures and modes of action (Figure 6). However, our data do not address the mechanism by which HCO_3_^−^ eliminates the effect of genistein on GABA_A_R/ATPase function. Given that HCO_3_^−^ has specific properties, including the regulation of neuronal activity Via interactions with cAMP or protein kinases [40], these findings suggest that HCO_3_^−^ can bind directly to amino acid residues and thereby modulate their conformation. This hypothesis is supported by data on direct interactions of HCO_3_^−^ with chemosensitive neurons and subsequent changes in their functional activity [40]. A detailed study of the phenol-sensitive mechanism by which isoflavones can interact directly with GABA_A_Rs will contribute to the design of new pharmacological agents.

## 4. Materials and Methods

### 4.1. Animals

Animal experiments were carried out using adult male Wistar rats purchased from the Institute of General Pathology and Pathophysiology vivarium and weighing 130–160 g at the time of arrival unless otherwise stated. Rats were always group-housed (5 per cage) and maintained in a temperature-controlled environment (23 ± 1) on a 12:12 h light-dark cycle and had access to food and water ad libitum. 

### 4.2. Synaptoneurosomes and Plasma Membrane Preparation

Synaptoneurosomes (SNs) and plasma membranes (PMs) were prepared from freshly dissected rat forebrains (cortex) or HEK 293FT cells as previously described.

### 4.3. Cell Cultures and Transfection

For the expression of homo- or heteromeric GABA_A_R ensembles, human embryonic kidney 293FT cells (American Type Culture Collection) were used. The cells were purchased from Invitrogen (Carlsbad, CA, USA) as part of the MembraneProTM Functional Protein Expression System (A11667), and the cell line identity was not further authenticated. The cells were grown and maintained in an incubator (Sanyo, Osaka, Japan) at 37 °C in a humidified atmosphere with 5% CO_2_, in DMEM media (41965-039, Gibco, Inchinnan, UK) supplemented with 0.1 mM MEM NEAA (11140035, Gibco, Inchinnan, UK), 4 mM l-glutamine, 1 mM sodium pyruvate, 4.5 g/L d-glucose (15023021), and 10% FBS (10270-106, Gibco, Waltham, MA, USA) until the 20th passage, as suggested by the vendor. HEK 293FT cells were transfected by Lipofectamine TM 2000 or 3000 (Invitrogen, Thermo Fisher Scientific, Waltham, MA, USA and Lithuania) transfection reagents according to the manufacturer’s instructions. Cells were harvested and analyzed 24 h after transfection. For transfection procedures and virus-like particle (VLP) production, the same growth medium with decreased FBS content up to 4% was used according to the manufacturer’s recommendations. Geneticin G418 sulfate (11811031, Invitrogen, Waltham, MA, USA) was present in the growth medium at a concentration of 500 mg/mL constantly except during the transfection. The cells were subcultured at confluence by treatment with 0.05% trypsin and 0.02% EDTA in PBS. For selection purposes and improving the yield of VLPs, the transfection medium was removed after 24 h, and a fresh growth medium with 10 µg/mL blasticidin (R21001 Gibco, Waltham, MA, USA) was added. Transfected cells and VLPs were collected and analyzed 24–48 h after transfection.

### 4.4. Molecular Biology

The genes encoding the full-length rat GABA_A_R α2, β3, or γ2 subunits were amplified by PCR from the cDNA library (Evrogen, Moscow, Russia) using gene-specific primers with Kozak sequence at the 5′ end of the forward primer based on ‘GenBank:NM_001135779.2’, ‘GenBank:NM_X15468.1’, and ‘GenBank:NM_183327.1’ sequences, respectively. The PCR products were cloned into the pEF6/V5-His TOPO TA vector (K961020, Invitrogen, Waltham, MA, USA) separately and verified by DNA sequencing. Each vector was amplified using *E. coli* TOP10 strain in LB medium supplemented with 20 µg/mL ampicillin. Isolation and purification of plasmids were performed with the PureYieldTM Plasmid Miniprep System (Promega, Madison, WI, USA) and Plasmid Midiprep 2.0 (Evrogen, Moscow, Russia). The sterilization of plasmids was implemented Via 0.22-µm filtration. The concentration of plasmids was evaluated on a spectrophotometer NanoDrop 1000 (Thermo Fisher Scientific, Waltham, MA, USA). The quality validation of cloning and growth was performed additionally through enzymatic restriction by XbaI and BamHI in BamHI buffer (Thermo Fisher Scientific, Waltham, MA, USA), and the following electrophoresis in 1% agarose gel.

The typical transfection procedure of GABA_A_R subunit-containing constructs for the subsequent biochemical, spectrofluorometric, and Western blot analyses was as follows. Approximately 5 × 105 HEK293FT cells were suspended in 8 mL DMEM, plated into a 90 mm culture dish, and maintained for approximately 24 h until 50–90% confluence. Then, 5 μg of plasmid DNA (β3 alone) was added combined with Lipofectamine^®^ 3000 Reagent (Invitrogen, Thermo Fisher Scientific, Waltham, MA, USA) in Opti-MEM^®^ I (1×) + GlutaMAXTM-I medium (51985-026, Gibco, Inchinnan, UK) according to the manufacturers’ recommendations. For microscopy, the cells were plated in 35 mm dishes and were incubated with a proportional amount of reagents and vectors.

For VLP production, GABA_A_R subunit-containing constructs were transfected together with Membrane Pro TM Reagent (Invitrogen, Waltham, MA, USA) amenably. Transfected HEK293FT started to bud off VLPs from the cell membrane approximately 24 h after transfection. The harvesting procedure was executed in conformity with the manufacturer’s recommendations. Briefly, the VLP-containing medium was mixed with Membrane Pro^TM^ Precipitation Mix in the ratio of 5 to 1, where 5 refers to the medium. Then, the mix was incubated at 4 °C overnight. After incubation, VLPs were pelleted by centrifugation at 5500× *g* for 30 min and resuspended in HEPES buffer for subsequent analysis or stored at −80 °C.

### 4.5. GABA_A_R-Mediated Cl^−^/HCO_3_^−^ Transport Monitoring

Cl^−^-sensitive fluorescent dye MQAE (N-(Ethoxycarbonyl-methyl)-6-Methoxyquinolinium Bromide) or pH-sensitive fluorescent dye BCECF, AM (2′,7′-Bis-(2-Carboxyethyl)-5-(and-6)-Carboxyfluorescein, Acetoxy methyl Ester) were obtained from Thermo Fisher Scientific, USA (E3101) or B1170, respectively. A stock solution was prepared in H_2_O, aliquoted, and stored in the freezer (−20 °C) protected from light. For cases in which fluorescence measurements were conducted, the HEK 293FT cells or SNs with loaded dye were stored in an opaque test tube at RT or 4 °C. Sodium bicarbonate (144-55-8), γ-aminobutyric acid, GABA (56-12-2), bicuculline (485-49-4), genistein (446-72-0) and (13721-39-6) were obtained from Sigma-Aldrich (St. Louis, MI, USA). SNs were loaded with dye (MQAE or BCECF) in BSS for 1 h at 37 °C and stored in an opaque test tube at RT or 4 °C. For that, the control HEK 293FT cells and GABA_A_R β3 isoform cells were trypsinized by adding 0.05% trypsin-EDTA solution (25200056, Gibco BRL, Waltham, MA, USA), washed with PBS twice, resuspended in the BSB, and then loaded with MQAE for 1 h at 37 °C. After loading, the suspension was centrifuged at 200× *g* for 5 min at RT and kept in the aforementioned medium at RT in the opaque test tubes. For analysis, the pellet was resuspended in the BSB. Monitoring was performed with cells continuously superfused with incubation medium composed of (mM):135 NaCl (or 135 NaCl and 25 NaHCO_3_), 0.5 KH_2_PO_4_, 0.8 MgCl_2_, and 5 mM Hepes (pH 7.4). Dye-loaded cells (SNS or HEK 293FT) were equilibrated in the test tube in the incubation medium (V = 200 µL) in the absence or presence of compounds for about 10 min at 37 °C before initial fluorescence measurements, and then 150 μL of the suspension was added into a quartz microcuvette (non-flow cell) and stirred. The GABA-mediated Cl^−^ or HCO_3_^−^ transport was initiated directly by the addition of GABA in a final concentration of 1–100 µM in the cuvette by an in-house solution supply system. GABA_A_R-mediated Cl^−^ or HCO_3_^−^ transport was assessed by the dynamic measurements of the variations in the fluorescence intensity of Cl^—^sensitive fluorescent dye MQAE-loaded or BCECF-loaded HEK 293FT cells or SNs using a FluoroMax^®^-4 spectrofluorometer (HORIBA Scientific Edison, Piscataway, NJ, USA), respectively. The excitation and emission wavelengths were 350 nm and 480 nm for the measurement of Cl^−^ transport or 490 nm and 535 nm for the measurement of HCO_3_^−^ transport, respectively. The Δ*F*/*F* of each trial was calculated as (*F*−*F*_0_)/*F*_0_, where *F*_0_ is the baseline fluorescence signal averaged over a 10 s period (this was the control measurement) immediately before the start of the application of GABA and supplement compounds. The value of 100% was obtained as the fluorescence intensity before the application of GABA (it was considered as non-specific change in fluorescence), in the absence or presence of test compounds. The maximum amplitude of GABA-mediated fluorescence responses was calculated as the maximal difference in fluorescence intensity in the absence or presence of an agonist. In addition, we performed several tests to assess whether cell integrity was maintained during the experiments (Supplementary Data).

Bicarbonate in the buffer was determined by an acid-base titration, where the sample is titrated against a standard acid solution (H_2_SO_4_) using phenolphthalein and methyl orange. Phenolphthalein is added as an indicator to detect the presence of carbonates (which will turn the solution pink). Methyl orange is then added to detect the bicarbonate. The solution will turn yellow, and the titration continues until it changes to orange, indicating the end point.

### 4.6. ATPase Activity Monitoring

The ATPase activity in PMs of neurons or HEK 293FT cells expressing the various constructs was measured as previously described [22]. PMs (~8 µg) or VLPs (~16 µg) were added into a glass tube with 0.5 mL of an incubation medium containing 20 mM HEPES-Tris pH 7.3, 5 mM NaCl/25 mM NaHCO_3_ (or 25 mM NaCl), 40 mM NaNO_3_ (neutral salt) and 0.5 mM EGTA and/or 50 µM ruthenium red (to eliminate the effect of Ca^2+^ATPase) to measure enzyme activity. The PMs or VLPs were preincubated at 37 °C for ~20 min with the relevant compounds in the incubation medium. Preparation of the test tube with bicarbonate–NaHCO_3_ (1 mM) was previously dissolved in HEPES (20 mM) and then added to the 20 mM HEPES-Tris buffer (pH 6.7). After preincubation, the enzyme reaction was started by the addition of Mg^2+^-ATP 2 mM (final concentration) in the incubation medium. After 15–20 min of incubation, the ATPase activity was stopped by the addition of reagents for measuring inorganic phosphorus (P_i_). The Cl^−^- and Cl^−^, HCO_3_^−^ATPase activities were determined as a difference in the formation of P_i_ in the absence and in the presence of NaCl (2–60 mM) or NaHCO_3_ (2.5–25 mM) in the incubation medium, respectively. Adenosine 5′-triphosphate (ATP) disodium salt hydrate (34369-07-8) and adenosine 5′-triphosphate disodium salt hydrate (A26209) were obtained from Merck (Kenilworth, NJ, USA). The concentration of P_i_ in the incubation medium was measured by a modified method of Chen et al. (1956) [15,22] using a Cary 60 UV–vis spectrophotometer (Agilent, Santa Clara, CA, USA) at a wavelength of 650 nm. The γ-phosphate analog, orthovanadate (VO_4_^3−^) (Sigma-Aldrich, St. Louis, MI, USA), was obtained by boiling the vanadate solution (pH 10; 10 min), and freshly boiled stock was diluted to the final concentration (pH 7.3) prior to use.

### 4.7. Protein Phosphorylation

The plasma membranes were phosphorylated in 30 μL of incubation medium containing 25 mM MOPS–Tris (pH 6.0), 3 mM MgSO_4_, and protein (approximately 40 μg). The phosphorylation reaction was started by the addition to the incubation medium of 70 μM ATP-γ-^32^P (specific radioactivity, 5 × 10^−6^ dpm/nmol) (Amersham, Biosciences, London, UK). The mixture was incubated at 0–1 °C for 2 min. To study the effect of 5 mM Cl^−^/25 mM HCO_3_^−^, genistein, vanadate or phenol on the phosphoprotein formation, the membrane preparation was preincubated with the ligands at 0–1 °C for 15 min.

### 4.8. Detection of the Phenol-Induced Behavioral Responses

Behavioral testing was conducted in a clear Plexiglas box (35 × 35 × 25 cm). On the day of experimentation, the animals were transferred to the testing room and left for 1 h for acclimatization. They were randomly assigned to experimental groups, and experimental HTR and tremors were produced in animals (the total number was 50 rats) by a single injection of phenol intraperitoneally (i.p.) as previously described [15]. In head twitching responses (HTR) and tremor tests, rats were divided into eight groups of five animals each. Group 1 (control) was treated with vehicle (NaCl 0.9%) i.p. Immediately after injections, animals returned to the monitoring chamber. HTR and tremor were recorded manually for 15 min post-injections. The number of animals in experimental groups regarding time points was five. The time period of analysis was selected from previous experiments showing that phenol-induced disorders occurred mainly within 15 min. HTR was defined as a rapid rhythmic shaking of the head in a radial motion. The tremor was assessed as the involuntary quivering movement of the whole body. To evaluate tremor, animals were placed individually in an observation box. The occurrence of tremors was rated by an observer blinded to the treatment protocol. The period between the injection of phenol and the appearance of the first symptoms of tremors was recorded at the time of onset of tremors. The duration of tremors was recorded as the time between onset and complete disappearance of tremors. Tremor intensity was evaluated using a four-point ranked scale: no tremor—0, mild tremor—1, moderate intermittent tremor—2, moderate persistent tremor—3, and pronounced severe tremor—4 [15].

### 4.9. Western Blot Analysis

Western blot was carried out as previously described [22]. The plasma membranes or VLPs of transfected 293FT cells, as well as the His-tagged purified receptor elution fractions, were subjected to SDS/PAGE using the SDS-PAGE Reagent Starter Kit (1615100 Bio-Rad, Hercules, CA, USA) and to Western blot analysis by the Pierce™ Fast Western Blot Kit (35055 Thermo Scientific) and the ECL Plus Western Blotting Detection System Substrate (GE Healthcare, Chicago, IL, USA). Samples were SDS-treated using boiling for 5 min in a buffer consisting of 62.5 mm Tris, 10% glycerol, 5% 2-mercaptoethanol, 4% SDS, and 0.001% bromophenol blue, and then ~20 µg of total protein was loaded into SDS/PAGE. Electrophoresis’ parameters were as follows: 70 V for 10 min on 4% SDS/PAGE stacking gel and 120 V for 50 min on 12.5% SDS/PAGE resolving gel. Proteins were transferred onto the PVDF membrane by the semidry method using 0.09 A·cm^−2^ for 1 h. After that, membranes were incubated for 1 h in a blocking solution containing 5% milk and then incubated at 4 °C overnight with primary GABRB3 monoclonal antibodies (MA5-27698 Thermo Fisher Scientific), diluted 1:2000 with the blocking solution. After incubation, the membranes were washed with TBS-T four times for 15 min each washing and then incubated at RT for 1 h with secondary HRP-conjugated antibodies (62-6520 Thermo Fisher Scientific), diluted 1:5000 with the blocking solution. Then, the membrane was washed with TBS-T four times, and the GE Healthcare ECL Plus Western Blotting Detection System (Amersham™, GE Healthcare) was applied according to the manufacturer’s instructions. The visualization of the bands was obtained on a Kodak Image Station 440 (Kodak, Rochester, NY, USA).

### 4.10. Statistical Analysis

The data are expressed as the mean ± SEM, and differences were considered significant for *p* < 0.05. Statistical differences were determined by a two-tailed Student’s unpaired *t*-test for data with equal variances and which were assessed as normally distributed with the Shapiro–Wilk test. Graphs and statistical analysis were obtained by using GraphPad Prism 9.3 (GraphPad Software, San Diego, CA, USA).

## Data Availability

The data presented in this study are available on request from the corresponding author.

## Supplementary Materials

The following supporting information can be downloaded at https://www.mdpi.com/article/10.3390/ijms26125731/s1.

## References

[1] Khan R., Kulasiri D., Samarasinghe S. (2021). Functional repertoire of protein kinases and phosphatases in synaptic plasticity and associated neurological disorders. Neural Regen. Res..

[2] Woolfrey K.M., Dell’Acqua M.L. (2015). Coordination of protein phosphorylation and dephosphorylation in synaptic plasticity. J. Biol. Chem..

[3] Hu H., Howard R.J., Bastolla U., Lindahl E., Delarue M. (2020). Structural basis for allosteric transitions of a multidomain pentameric ligand-gated ion channel. Proc. Natl. Acad. Sci. USA.

[4] Menzikov S.A., Zaichenko D.M., Moskovtsev A.A., Morozov S.G., Kubatiev A.A. (2024). Phenols and GABA_A_ receptors: From structure and molecular mechanisms action to neuropsychiatric sequelae. Front. Pharmacol..

[5] Jassar B.S., Ostashewski P.M., Jhamandas J.H. (1997). GABA_A_ receptor modulation by protein tyrosine kinase in the rat diagonal band of Broca. Brain Res..

[6] Minier F., Laschet J.J., Evrard B., Bureau M.H. (2000). Endogenous phosphorylation of the GABA(A) receptor protein is counteracted by a membrane-associated phosphatase. Neurochem. Int..

[7] Castel H., Louiset E., Anouar Y., Le Foll F., Cazin L., Vaudry H. (2000). Regulation of GABA_A_ receptor by protein tyrosine kinases in frog pituitary melanotrophs. J. Neuroendocrinol..

[8] Wan Q., Man H.Y., Braunton J., Wang W., Salter M.W., Becker L., Wang Y.T. (1997). Modulation of GABA_A_ receptor function by tyrosine phosphorylation of beta subunits. J. Neurosci..

[9] Moss S.J., Gorrie G.H., Amato A., Smart T.G. (1995). Modulation of GABAA receptors by tyrosine phosphorylation. Nature.

[10] Menzikov S.A., Zaichenko D.M., Moskovtsev A.A., Morozov S.G., Kubatiev A.A. (2021). Ectopic GABA_A_ receptor β3 subunit determines Cl^−^/HCO_3_^−^-ATPase and chloride transport in HEK 293FT cells. FEBS J..

[11] Dunne E.L., Moss S.J., Smart T.G. (1998). Inhibition of GABA_A_ receptor function by tyrosine kinase inhibitors and their inactive analogues. Mol. Cell Neurosci..

[12] Huang R.Q., Fang M.J., Dillon G.H. (1999). The tyrosine kinase inhibitor genistein directly inhibits GABA_A_ receptors. Brain Res. Mol. Brain Res..

[13] Starovoytov O.N., Liu Y., Tan L., Yang S. (2014). Effects of the hydroxyl group on phenyl based ligand/ERRγ protein binding. Chem. Res. Toxicol..

[14] Tracey A.S., Gresser M.J. (1986). Interaction of vanadate with phenol and tyrosine: Implications for the effects of vanadate on systems regulated by tyrosine phosphorylation. Proc. Natl. Acad. Sci. USA.

[15] Menzikov S.A., Morozov S.G. (2019). Involvement of brain GABA_A_R-coupled Cl^−^/HCO_3_^−^-ATPase in phenol-induced the head-twitching and tremor responses in rats. Neurotoxicology.

[16] Brosnan R.J., Pham T.L. (2018). Anesthetic-sensitive ion channel modulation is associated with a molar water solubility cut-off. BMC Pharmacol. Toxicol..

[17] Ghit A., Assal D., Al-Shami A.S., Hussein D.E.E. (2021). GABA_A_ receptors: Structure, function, pharmacology, and related disorders. J. Genet. Eng. Biotechnol..

[18] Kim D.Y., Fenoglio K.A., Kerrigan J.F., Rho J.M. (2009). Bicarbonate contributes to GABA_A_ receptor-mediated neuronal excitation in surgically resected human hypothalamic hamartomas. Epilepsy Res..

[19] Lombardi A., Jedlicka P., Luhmann H.J., Kilb W. (2019). Interactions between Membrane Resistance, GABA-A Receptor Properties, Bicarbonate Dynamics and Cl^−^-Transport Shape Activity-Dependent Changes of Intracellular Cl^−^-Concentration. Int. J. Mol. Sci..

[20] Sigel E., Steinmann M.E. (2012). Structure, function, and modulation of GABA_A_ receptors. J. Biol. Chem..

[21] Menzikov S.A., Morozov S.G., Kubatiev A.A. (2021). Intricacies of GABAA Receptor Function: The Critical Role of the β3 Subunit in Norm and Pathology. Int. J. Mol. Sci..

[22] Menzikov S.A., Zaichenko D.M., Moskovtsev A.A., Morozov S.G., Kubatiev A.A. (2023). Zinc Inhibits the GABA_A_R/ATPase during Postnatal Rat Development: The Role of Cysteine Residue. Int. J. Mol. Sci..

[23] Menzikov S.A., Zaichenko D.M., Moskovtsev A.A., Morozov S.G., Kubatiev A.A. (2022). Physiological Role of ATPase for GABA_A_ Receptor Resensitization. Int. J. Mol. Sci..

[24] Elsayed A.A., Menze E.T., Tadros M.G., Ibrahim B.M.M., Sabri N.A., Khalifa A.E. (2018). Effects of genistein on pentylenetetrazole-induced behavioral and neurochemical deficits in ovariectomized rats. Naunyn Schmiedebergs Arch. Pharmacol..

[25] Hu Q.P., Yan H.X., Peng F., Feng W., Chen F.F., Huang X.Y., Zhang X., Zhou Y.Y., Chen Y.S. (2021). Genistein protects epilepsy-induced brain injury through regulating the JAK2/STAT3 and Keap1/Nrf2 signaling pathways in the developing rats. Eur. J Pharmacol..

[26] Westmark C.J. (2014). A hypothesis regarding the molecular mechanism underlying dietary soy-induced effects on seizure propensity. Front. Neurol..

[27] Jin Y., Wu H., Cohen E.M., Wei J., Jin H., Prentice H., Wu J.Y. (2007). Genistein and daidzein induce neurotoxicity at high concentrations in primary rat neuronal cultures. J. Biomed. Sci..

[28] Guo J., Min D., Feng H.J. (2021). Genistein, a Natural Isoflavone, Alleviates Seizure-Induced Respiratory Arrest in DBA/1 Mice. Front. Neurol..

[29] Sallard E., Letourneur D., Legendre P. (2021). Electrophysiology of ionotropic GABA receptors. Cell Mol. Life Sci..

[30] Talwar S., Lynch J.W., Gilbert D.F. (2013). Fluorescence-based high-throughput functional profiling of ligand-gated ion channels at the level of single cells. PLoS ONE.

[31] Chua H.C., Absalom N.L., Hanrahan J.R., Viswas R., Chebib M. (2015). The Direct Actions of GABA, 2’-Methoxy-6-Methylflavone and General Anaesthetics at β3γ2L GABAA Receptors: Evidence for Receptors with Different Subunit Stoichiometries. PLoS ONE.

[32] Ruffin V.A., Salameh A.I., Boron W.F., Parker M.D. (2014). Intracellular pH regulation by acid-base transporters in mammalian neurons. Front. Physiol..

[33] Chang Y., Ghansah E., Chen Y., Ye J., Weiss D.S. (2002). Desensitization mechanism of GABA receptors revealed by single oocyte binding and receptor function. J. Neurosci..

[34] Svichar N., Esquenazi S., Chen H.Y., Chesler M. (2011). Preemptive regulation of intracellular pH in hippocampal neurons by a dual mechanism of depolarization-induced alkalinization. J. Neurosci..

[35] Ríos J.L., Schinella G.R., Moragrega I. (2022). Phenolics as GABA_A_ Receptor Ligands: An Updated Review. Molecules.

[36] Khodamoradi M., Ghazvini H., Esmaeili-Mahani S., Shahveisi K., Farnia V., Zhaleh H., Abdoli N., Akbarnejad Z., Saadati H., Sheibani V. (2018). Genistein attenuates seizure-induced hippocampal brain-derived neurotrophic factor overexpression in ovariectomized rats. J. Chem. Neuroanat..

[37] Folarin R.O., Surajudeen O.B., Omotosho E.O., Surajudeen O.B., Oyeleye O.D.O., Shallie P. (2020). Motor co-ordinative roles of Nigella sativa oil in mice models of phenol-induced essential tremor. Ann. Health Res..

[38] Kang Y.J., Zuo L.M., Luo S.Z. (2010). Hydrogen-Bonding Interactions Induced by Phosphorylation Influence the Local Conformation of Phosphopeptides. Int. J. Pept Res. Ther..

[39] León J., Millán E.J., Cocinero A.L., Castaño F., José A.F. (2012). Mimicking anaesthetic–receptor interaction: A combined spectroscopic and computational study of propofol⋯phenol. Phys. Chem. Chem. Phys..

[40] Gonçalves C.M., Mulkey D.K. (2018). Bicarbonate directly modulates activity of chemosensitive neurons in the retrotrapezoid nucleus. J. Physiol..

