# Supplementary material for: Genistein and Vanadate Differentially Modulate Cortical GABAA Receptor/ATPase Activity and Behavior in Rats via a Phenol-Sensitive Mechanism
Source: Int J Mol Sci. 2025 Jun 15;26(12):5731. doi: 10.3390/ijms26125731 (PMC12193411; doi:10.3390/ijms26125731)
Supplement: Supplementary file 1 [file ijms-26-05731-s001.zip › ijms-3638837-supplementary.pdf]

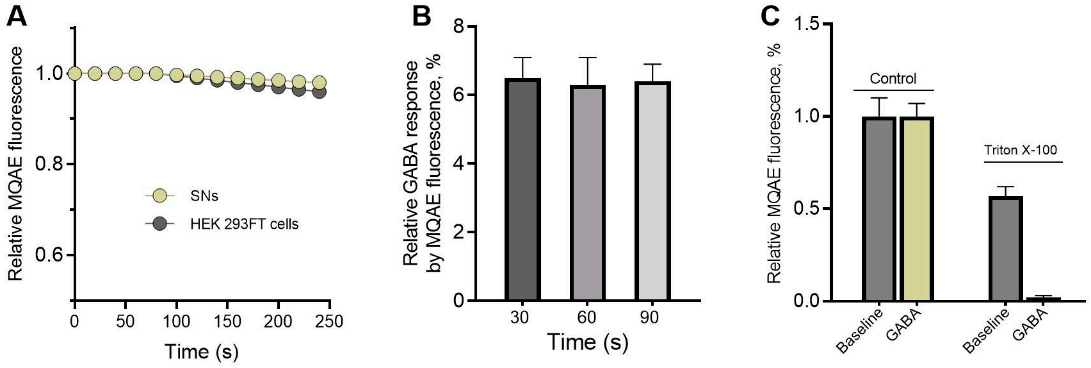

Figure 1. Relative MQAE fluorescence intensity indicates no major dye leakage in SNs or HEK 293FT cells. (**A**) Corresponding temporary MQAE fluorescence changes for SNs or HEK 293FT cells. (**B**) Similar MQAE fluorescence changes at GABA action during different times indicate something about maintaining the integrity of cells. (**C**) The validity of this approach is supported by the observation from experiments with cells that final fluorescence was the same when 200 μM Triton X-100 (1 %) was used to destroy the permeability of plasma membranes and equilibrate extra- and intracellular chloride.
